# Body Composition and Thermographic Asymmetry in Older Adults: A Cross-Sectional Study

**DOI:** 10.3390/healthcare14152295

**Published:** 2026-07-29

**Authors:** Sandra Núñez-Rodríguez, Marina Ortega-Santamaría, Félix Menéndez-Vega, Carla Collazo-Riobó, Sergio Alvarez-Pardo, Anna Berardi, Josefa González-Santos

**Affiliations:** 1 Faculty of Health Sciences, University Isabel I, 09003 Burgos, Spain; sandra.nunez@ui1.es (S.N.-R.); marina.ortega@ui1.es (M.O.-S.); felix.menendez@ui1.es (F.M.-V.); 2 Department of Human Neurosciences, University of la Sapienza, 00188 Rome, Italy; ccollazo@ubu.es (C.C.-R.); anna.berardi@uniroma1.it (A.B.); 3 Faculty of Health Science, University of Burgos, 09001 Burgos, Spain; mjgonzalez@ubu.es

**Keywords:** infrared thermography, body composition, aging, body fat percentage, thermal asymmetry, older adults

## Abstract

**Background/Objectives:** Aging is associated with physiological changes that impair thermoregulation and may increase vulnerability to thermal stress. Body composition, particularly adiposity and skeletal muscle mass, may influence skin temperature distribution and thermographic asymmetry; however, evidence in older adults remains limited. This study aimed to characterize thermographic asymmetry patterns in older adults and examine their association with body composition parameters potentially influencing thermoregulatory function during aging. **Methods**: A cross-sectional study was conducted in 127 community-dwelling older adults from Burgos, Spain. Body composition was assessed using multi-frequency bioelectrical impedance analysis (InBody S10), including the body fat percentage (PBF), appendicular skeletal muscle mass index (ASMI), phase angle, and extracellular water-to-total body water ratio (ECW/TBW). Infrared thermography was performed using an FLIR E6390 thermal camera (FLIR Systems Inc., Wilsonville, OR, USA), and thermographic images were analyzed with ThermoHuman software version 3.0. Variables included global temperature, trunk thermal asymmetry, upper-limb thermal asymmetry, mean absolute thermal asymmetry, the Thermal Recovery Index (TRI), and the Symmetry Assessment Parameter (SAP). Sex differences, Spearman correlations, and multiple linear regression analyses were performed. **Results**: Women presented significantly higher body fat percentage and greater thermographic asymmetry than men (*p* < 0.05). Body fat percentage was positively associated with upper-limb thermal asymmetry (ρ = 0.229, *p* = 0.015), mean absolute thermal asymmetry (ρ = 0.280, *p* = 0.003), TRI (ρ = 0.250, *p* = 0.008), and SAP (ρ = 0.276, *p* = 0.003). In contrast, the ASMI, phase angle, and ECW/TBW ratio showed no significant associations with thermographic asymmetry indices. In the adjusted regression analysis, body fat percentage showed a statistically significant coefficient for mean absolute thermal asymmetry; however, the overall model was not statistically significant and explained only 5% of the variance (R^2^ = 0.050, *p* = 0.144). **Conclusions**: Body fat percentage showed modest associations with several thermographic asymmetry parameters, whereas skeletal muscle mass showed limited relationships with thermographic outcomes. Given the weak magnitude of the correlations and the limited explanatory capacity of the regression model, these findings should be considered exploratory. Body composition may represent one of several factors associated with thermographic variability in older adults.

## 1. Introduction

Population ageing is accompanied by physiological changes that may compromise thermal regulation and increase vulnerability to environmental thermal stress. Older adults show reduced efficiency in heat dissipation and thermal adaptation due to age-related changes in cutaneous blood flow, cardiovascular control, sweating responses, autonomic regulation, and metabolic heat production [1,2]. These alterations are clinically relevant because impaired thermoregulation has been associated with functional decline, impaired physiological adaptation to environmental temperature changes, and an increased risk of adverse events during heat or cold exposure in older adults [3,4].

Body composition is a key determinant of thermal physiology. Adipose tissue acts as an insulating layer and can influence heat storage, heat dissipation, and regional skin temperature patterns, whereas skeletal muscle contributes to resting energy expenditure and endogenous heat production [5,6]. In older adults, increased adiposity and reduced muscle mass are common and may affect peripheral temperature distribution and thermal symmetry. Although changes in body composition may influence whole-body heat transfer, adipose tissue distribution is not necessarily symmetrical between contralateral body regions. Regional differences in subcutaneous fat deposition, tissue insulation, local perfusion, and microvascular regulation may therefore contribute to asymmetric skin temperature patterns rather than producing homogeneous changes in skin temperature [5,7,8]. The revised European consensus on sarcopenia highlights the clinical importance of skeletal muscle deterioration in ageing, but the relationship between muscle-related parameters and thermographic asymmetry remains insufficiently characterized [9,10,11].

Infrared thermography is a non-invasive and contact-free technique that allows the assessment of skin temperature distribution and thermal asymmetry. Recent reviews have highlighted its potential clinical utility while emphasizing the need for rigorous standardization of image acquisition, environmental conditions, and interpretation procedures [12,13]. Consensus recommendations for human skin temperature assessment also stress that thermographic outcomes may be influenced by individual and contextual factors, including body composition, environmental exposure, and measurement protocol [14].

Previous studies have shown that adiposity and body fat distribution can influence skin surface temperature assessed by infrared thermography [7,15]. More recent evidence has also indicated that body mass index and body composition should be considered when interpreting thermographic images, as anthropometric and adiposity-related variables may affect skin temperature responses [16,17]. However, most available studies have focused on younger populations, athletes, or specific anatomical regions, while evidence in community-dwelling older adults remains limited [18,19,20]. In particular, little is known about whether adiposity and appendicular skeletal muscle mass are associated with global thermographic asymmetry patterns in older adults.

Recent studies have further highlighted the usefulness of infrared thermography for evaluating thermoregulatory responses in older adults. For example, Costa et al. demonstrated that thermal imaging can identify age-related differences in skin temperature distribution and emphasized its potential as a non-invasive tool for monitoring physiological responses in aging populations. Likewise, recent investigations have reinforced the importance of considering age-related thermoregulatory impairment when interpreting thermographic findings in older individuals, supporting the need for standardized assessment protocols and greater understanding of factors influencing thermal variability [21,22].

Thermoregulation requires the coordinated interaction of cardiovascular, autonomic, metabolic, and musculoskeletal systems, all of which may be compromised during aging [1,2]. Consequently, alterations in thermal symmetry and skin temperature distribution may reflect differences in thermoregulatory responses between individuals. Understanding the factors that influence thermographic patterns may therefore contribute to improving the interpretation of infrared thermography and support future research on thermoregulatory function in older adults [23,24].

Therefore, the aim of this study was to characterize thermographic asymmetry patterns in older adults and to analyze their association with body composition parameters, particularly body fat percentage and appendicular skeletal muscle mass index. A secondary objective was to explore potential sex-specific differences in body composition and thermographic parameters. Given the role of body composition in thermoregulation during aging, a better understanding of these relationships may contribute to the interpretation of thermographic asymmetry patterns in older adults. We hypothesized that higher adiposity and lower muscle mass would be associated with greater thermographic asymmetry in older adults.

## 2. Materials and Methods

### 2.1. Study Design and Participants

A cross-sectional observational study was conducted in older adults from Burgos (Castilla y León, Spain). Participant recruitment and data collection were carried out between February and April 2026. A convenience sampling strategy was used to recruit community-dwelling adults aged 60 years and older from community centers and social care facilities. Although the eligibility criterion was an age of ≥60 years, recruitment was primarily conducted in facilities serving older populations, resulting in a sample with a mean age of 81.0 ± 10.7 years.

Individuals who met the eligibility criteria were informed about the objectives and procedures of the study, and those who voluntarily agreed to participate and provided written informed consent were enrolled.

No eligibility criteria based on body composition (e.g., body fat percentage, body mass index, or skeletal muscle mass) were applied, as the aim was to investigate the association between body composition and thermographic variables across a heterogeneous sample of community-dwelling older adults. A priori sample size calculation was performed using GPower software (version 3.1.9.7; Heinrich Heine University Düsseldorf, Düsseldorf, Germany). As the primary objective was to examine the association between body composition and thermographic variables, the calculation was based on a two-tailed correlation analysis. Assuming an expected small-to-moderate correlation (*r* = 0.25), an alpha level of 0.05, and a statistical power of 80%, a minimum sample size of 124 participants was required. The final sample comprised 127 participants, thereby exceeding the estimated minimum sample size. Individuals presenting with fever, acute illness, active infection, abnormal vital signs, or any other clinical condition that could interfere with the assessment protocol were excluded. Participant eligibility was determined on the day of assessment following a routine clinical evaluation performed by the physician and nursing staff at the participating institution, which included the review of vital signs and general health status. Only participants considered clinically stable by the healthcare professionals were assessed.

Wheelchair use was not considered an exclusion criterion. Participants who used a wheelchair for mobility were eligible provided they were able to safely perform the required transfers, maintain the standing position during thermographic image acquisition, and complete all body composition assessments according to the standardized protocol.

All participants voluntarily agreed to participate and provided written informed consent prior to inclusion. The study was conducted in accordance with the ethical principles of the Declaration of Helsinki. Ethical approval was obtained from the Ethics Committee of Universidad Isabel I (protocol code Ui1-PI127—CEI25-05-PI127). All participants provided written informed consent prior to participation.

Variables included in the present analysis were selected based on physiological relevance and previous literature regarding thermoregulation, thermal asymmetry, and body composition in older adults.

For the purposes of this study, body composition parameters (body fat percentage, appendicular skeletal muscle mass index, phase angle, and extracellular water-to-total body water ratio) were considered the primary exposure variables. Thermographic parameters were considered the primary outcome variables. Age and sex were treated as potential confounding variables and were included as adjustment variables in the multiple linear regression analysis.

### 2.2. Body Composition Assessment

Body composition was assessed using a multi-frequency bioelectrical impedance analyzer (InBody S10, InBody Co., Seoul, Republic of Korea). The InBody S10 is a direct segmental multi-frequency bioelectrical impedance device that has demonstrated good validity and high test–retest reliability for body composition assessment in older adults when standardized measurement procedures are followed [25,26,27,28]. The variables included in the present study were body fat percentage (PBF), extracellular water-to-total body water ratio (ECW/TBW), phase angle (PhA), and appendicular skeletal muscle mass index (ASMI).

To minimize the influence of hydration status and other physiological factors known to affect bioelectrical impedance measurements, all assessments were performed under standardized conditions following the manufacturer’s recommendations.

Appendicular skeletal muscle mass (ASM) was calculated as the sum of lean mass from both upper and lower limbs obtained from the bioimpedance assessment. ASMI was subsequently calculated as ASM divided by height squared (kg/m^2^).

### 2.3. Thermographic Assessment

Infrared thermographic images were acquired using a FLIR E6390 thermal camera (FLIR Systems Inc., Wilsonville, OR, USA). Image processing and thermographic analyses were subsequently performed using ThermoHuman® software (version 3.0) and reviewed using FLIR Tools software (version 6.4). Infrared thermographic assessment was performed using a FLIR E6390 thermal camera (FLIR Systems Inc., Wilsonville, OR, USA). Thermal image acquisition was performed using a FLIR E6390 thermal camera. Image processing and thermographic analysis were conducted using ThermoHuman^®^ software (version 3.0), and all images were subsequently reviewed using FLIR E6390 thermal camera (FLIR Systems Inc., Wilsonville, OR, USA). All thermographic evaluations were performed in a controlled room at 22 ± 1 °C under standardized environmental conditions. Participants underwent a 15 min acclimatization period prior to image acquisition. Thermal images were obtained at a standardized distance of 1.5 m with participants in an anatomical standing position and wearing only underwear to minimize interference with skin temperature assessment.

Participants were instructed to avoid intense physical activity, caffeine, alcohol, and smoking for at least 12 h prior to assessment. Environmental conditions were maintained as stable as possible throughout all evaluations.

**Figure 1:**
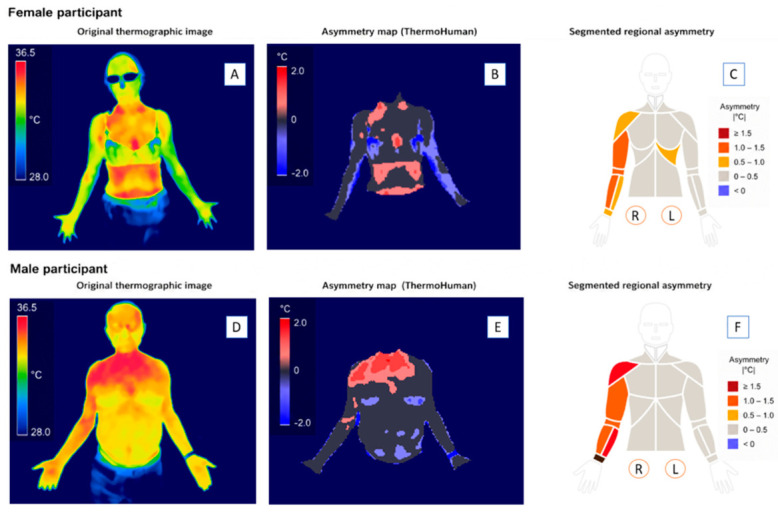
Representative thermographic image-processing workflow using ThermoHuman^®^ software (version 3.0). Examples from a female participant (**A**–**C**) and a male participant (**D**–**F**). Original thermographic images (**A**,**D**), asymmetry maps (**B**,**E**), and segmented regional asymmetry analyses (**C**,**F**) are shown. Warmer colors indicate greater thermal asymmetry between contralateral body regions.

Regions of interest were automatically identified using ThermoHuman^®^ software (version 3.0) following its validated automatic anatomical segmentation procedures, which identify homologous body regions and calculate regional skin temperature and bilateral thermal asymmetry using standardized image-processing algorithms [29]. The automated image-processing procedures implemented in the ThermoHuman platform have previously demonstrated acceptable validity and reproducibility for standardized thermographic analyses [29]. Analyses were performed using anterior body images, including trunk and bilateral upper-limb regions. Representative examples of the thermographic image-processing workflow, including the original thermograms, asymmetry maps, and automatically segmented regions of interest, are presented in Figure 1.

### 2.4. Thermographic Variables

The thermographic variables analyzed in this study were selected to characterize global skin temperature distribution and thermal asymmetry patterns.

Global temperature represented the overall mean skin temperature of the body surface. Trunk thermal asymmetry and upper-limb thermal asymmetry reflected temperature differences between contralateral body regions, providing information on bilateral thermal balance.

Mean absolute thermal asymmetry represented the average magnitude of temperature differences between contralateral body regions identified by the software and was used as an indicator of overall thermal imbalance. Higher values reflected greater asymmetry in skin temperature distribution between homologous regions.

The Symmetry Assessment Parameter (SAP) was used as a composite indicator of bilateral thermal symmetry derived from the comparison of homologous contralateral body regions identified by the ThermoHuman software (version 3.0). Higher SAP values indicate greater bilateral thermal symmetry across the analyzed regions. The Thermal Recovery Index (TRI) was included as a complementary composite thermographic indicator generated automatically by the software, reflecting the overall thermographic response based on the analyzed thermal patterns. Together with mean absolute thermal asymmetry, these indices were selected because they provide complementary information regarding global thermographic behaviour beyond isolated regional skin temperature measurements.

### 2.5. Statistical Analysis

Statistical analyses were performed using IBM SPSS Statistics version 28.0 (IBM Corp., Armonk, NY, USA). The normality of the variables was assessed using the Shapiro–Wilk test. Analyses were performed using complete-case data for the variables included in each statistical procedure. Consequently, the number of participants varied slightly across analyses depending on data availability. Consequently, all analyses were performed using complete-case data from the final study sample.

Descriptive statistics were expressed as mean ± standard deviation. Differences between men and women were analyzed using the Mann–Whitney U test. Associations between thermographic and body composition variables were evaluated using Spearman’s rank correlation coefficient.

A multiple linear regression analysis was performed to examine the independent association between the primary exposure variable (body fat percentage) and the primary outcome variable (mean absolute thermal asymmetry), adjusting for the potential confounding effects of age and sex. Mean absolute thermal asymmetry was selected as the primary thermographic outcome because it provides a direct measure of the overall magnitude of bilateral thermal asymmetry across the analyzed body regions and best reflected the primary objective of the study. The TRI and SAP were considered complementary composite thermographic indices and were therefore evaluated as exploratory outcomes using correlation analyses. Body fat percentage was entered into the model as the primary exposure variable because it showed the most consistent associations with the thermographic outcomes, whereas age and sex were included as potential confounding variables. Prior to model fitting, the assumptions of linear regression were assessed. Multicollinearity was evaluated using variance inflation factors (VIFs) and tolerance values, whereas the normality, homoscedasticity, and independence of residuals were assessed through graphical inspection of standardized residuals and the Durbin–Watson statistic. No substantial violations of the regression assumptions were identified.

All statistical tests were two-tailed, and statistical significance was established at *p* < 0.05. Because the correlation analyses were exploratory in nature, no formal adjustment for multiple comparisons was applied. Consequently, statistically significant findings should be interpreted with appropriate caution.

## 3. Results

**Figure 2:**
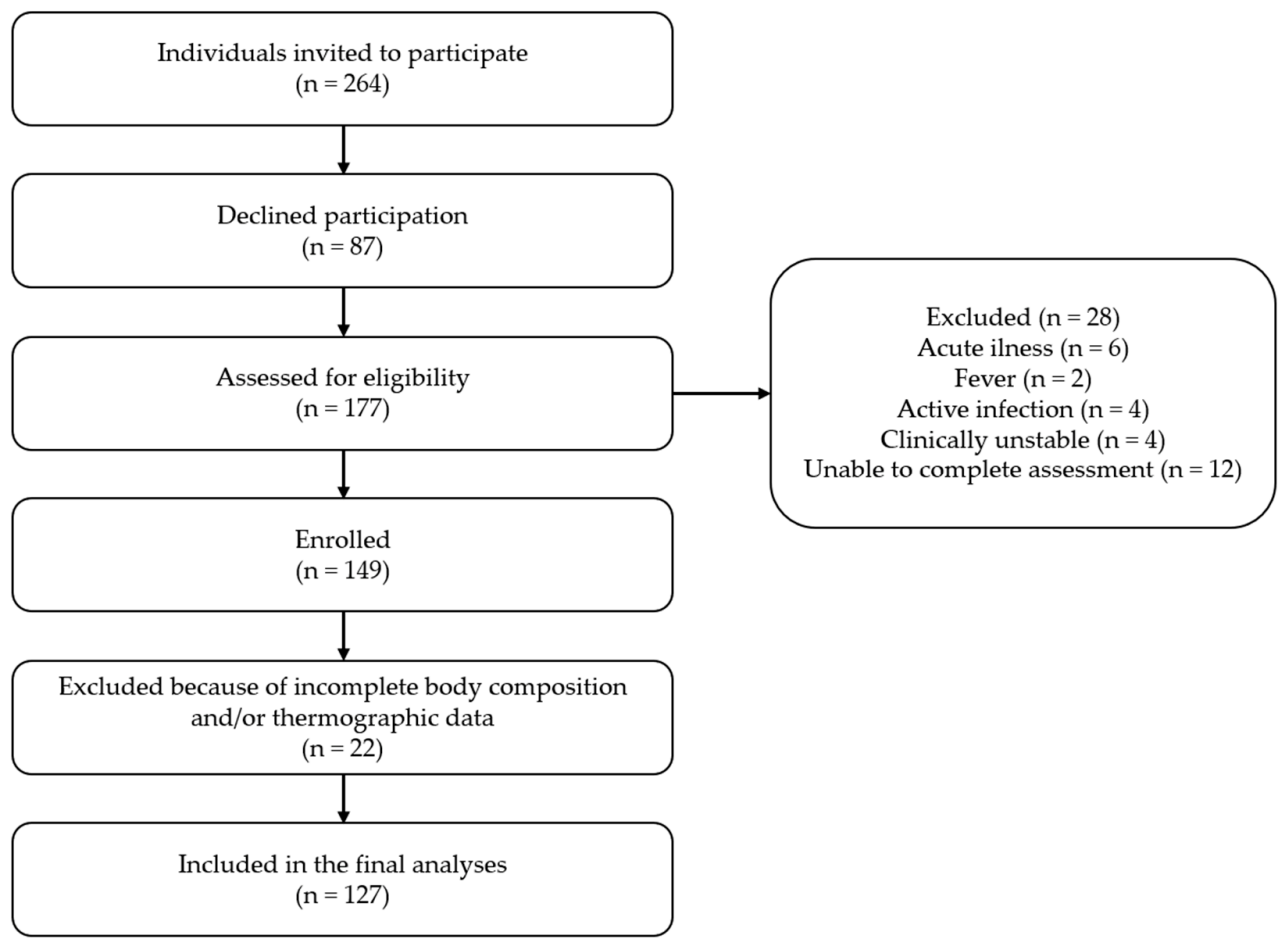
Participant flow diagram. Flowchart illustrating participant recruitment, eligibility assessment, exclusions, and inclusion in the final analyses. Participants were excluded because of acute illness, fever, active infection, clinical instability, inability to complete the assessment protocol, or incomplete body composition and/or thermographic data.

The participant selection process is summarized in Figure 2. A total of 264 community-dwelling older adults were invited to participate, of whom 87 declined participation. Following eligibility assessment, 28 individuals were excluded because of acute illness, fever, active infection, clinical instability, or inability to complete the assessment protocol. A total of 149 participants were enrolled. Of these, 22 were excluded before the statistical analyses because complete body composition and/or thermographic assessments were unavailable, primarily because previously unreported contraindications to bioelectrical impedance analysis (e.g., pacemaker implantation) were identified on the day of assessment. Consequently, the final analytical sample comprised 127 participants. The number of participants included in each statistical analysis varied according to the availability of the required variables.

### 3.1. Participant Characteristics

A total of 127 older adults were included in the study. The descriptive characteristics of the study sample are presented in Table 1.

Participants showed a mean age of 81.0 ± 10.7 years and a mean body mass index of 28.0 ± 5.7 kg/m^2^. Mean body fat percentage was 38.3 ± 11.5%, whereas the mean appendicular skeletal muscle mass index was 6.86± 1.95 kg/m^2^.

Regarding thermographic parameters, the mean global temperature was 33.20 ± 0.97 °C, and the mean absolute thermal asymmetry value was 0.46 ± 0.24.

### 3.2. Sex Differences

Sex-based comparisons are presented in Table 2. Women showed significantly higher body fat percentage values than men (40.4 ± 11.8% vs. 34.3 ± 9.2%, *p* < 0.001), whereas men presented significantly higher appendicular skeletal muscle mass index values (7.7 ± 1.9 vs. 6.4 ± 1.8 kg/m^2^, *p* < 0.001).

Regarding thermographic variables, women exhibited significantly higher trunk thermal asymmetry values compared with men (0.34 ± 0.14 vs. 0.28 ± 0.15, *p* = 0.006). Women also showed higher mean absolute thermal asymmetry, Thermal Recovery Index, and Symmetry Assessment Parameter values.

No significant differences between sexes were observed for global temperature, upper-limb thermal asymmetry, phase angle, or ECW/TBW ratio.

### 3.3. Correlations Between Thermographic and Body Composition Variables

Spearman correlation analyses between thermographic and body composition variables are presented in Table 3.

Body fat percentage showed the most consistent associations with thermographic asymmetry-related parameters. Specifically, higher body fat percentage values were associated with greater upper-limb thermal asymmetry (ρ = 0.229, *p* = 0.015), mean absolute thermal asymmetry (ρ = 0.280, *p* = 0.003), Thermal Recovery Index values (ρ = 0.250, *p* = 0.008), and Symmetry Assessment Parameter values (ρ = 0.276, *p* = 0.003). In contrast, appendicular skeletal muscle mass index, phase angle, and ECW/TBW ratio were not significantly associated with thermographic asymmetry indices.

In sex-stratified analyses, the association between body fat percentage and mean absolute thermal asymmetry remained significant in women (ρ = 0.296, *p* = 0.012), whereas no significant association was observed in men (ρ = 0.128, *p* = 0.433).

Given the relatively wide age range of the study population, exploratory univariate analyses were performed to assess the association between age and the thermographic parameters. No statistically significant correlations were observed between age and global temperature (ρ = 0.117, *p* = 0.212), trunk thermal asymmetry (ρ = 0.043, *p* = 0.648), upper-limb thermal asymmetry (ρ = 0.071, *p* = 0.449), mean absolute thermal asymmetry (ρ = 0.127, *p* = 0.176), TRI (ρ = 0.143, *p* = 0.126), or SAP (ρ = 0.052, *p* = 0.579).

**Figure 3:**
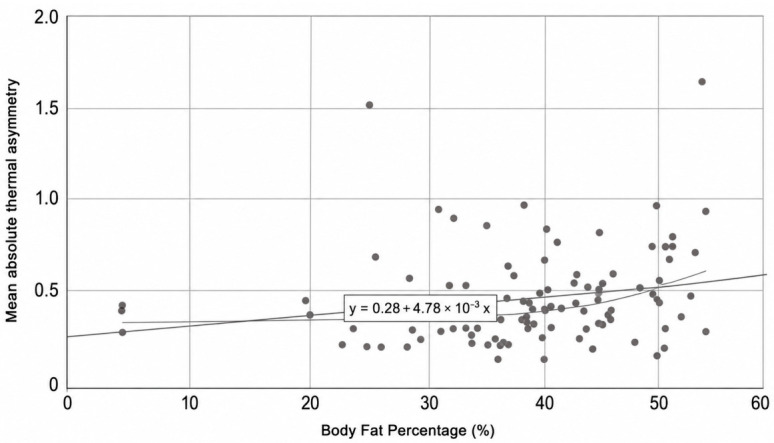
Scatterplot illustrating the association between body fat percentage (primary exposure variable) and mean absolute thermal asymmetry (primary outcome variable) in the study population. Mean absolute thermal asymmetry was selected as the overall thermographic measure and was subsequently included in the adjusted multiple linear regression analysis.

Because body fat percentage and mean absolute thermal asymmetry represented the primary exposure and primary outcome variables of the study, respectively, their association is illustrated in Figure 3. Mean absolute thermal asymmetry was selected because it provides an overall measure of bilateral thermal asymmetry and was the thermographic outcome included in the adjusted multiple linear regression analysis.

In contrast, appendicular skeletal muscle mass index did not show significant associations with thermographic asymmetry parameters. Skeletal muscle mass showed a weak negative correlation with trunk thermal asymmetry (ρ = −0.199, *p* = 0.035).

Additionally, the ECW/TBW ratio was positively associated with global temperature (ρ = 0.260, *p* = 0.005).

### 3.4. Multiple Linear Regression Analysis

Because body fat percentage was the only body composition variable consistently associated with the thermographic asymmetry indices in the univariate analyses, it was selected for inclusion in the adjusted multiple linear regression model (Table 4). Age and sex were retained in the model as prespecified adjustment variables because of their established physiological relevance to thermoregulation, regardless of their univariate associations with the outcome. In the adjusted multiple linear regression model, body fat percentage showed a statistically significant regression coefficient (B = 0.005; 95% CI: 0.001 to 0.009; *p* = 0.026), whereas age and sex were not significantly associated with mean absolute thermal asymmetry. However, the overall regression model did not reach statistical significance (F = 1.840, *p* = 0.144) and explained only a small proportion of the variance (R^2^ = 0.050). Therefore, these findings should be interpreted with caution.

## 4. Discussion

Body composition, particularly body fat percentage, showed weak but relatively consistent associations with several thermographic parameters in the present study. Specifically, higher body fat percentage was associated with mean thermal asymmetry, TRI, and SAP, whereas skeletal muscle mass showed considerably weaker relationships with thermographic outcomes. Although these associations reached statistical significance, their magnitude was generally weak, indicating that body composition explained only a limited proportion of the variability in thermographic patterns. Women exhibited higher thermal asymmetry than men in the unadjusted analyses. However, after adjustment for body fat percentage, sex was no longer independently associated with mean thermal asymmetry. Collectively, these findings suggest that adiposity should be considered as one of several factors associated with thermographic patterns in older populations rather than as the sole determinant of thermal asymmetry. The association between adiposity and thermal asymmetry is biologically plausible. Adipose tissue acts as an insulator with lower thermal conductivity than lean tissue and influences heat transfer between deeper tissues and the skin surface [5,7,18,20]. In addition, obesity has been associated with endothelial dysfunction, impaired microvascular responsiveness, and alterations in peripheral blood flow regulation, mechanisms that may contribute to heterogeneous skin temperature distributions and asymmetric heat dissipation [8,15,20,30]. These effects may become even more relevant with advancing age, as aging is accompanied by reduced thermoregulatory efficiency, diminished vasomotor responsiveness, and impaired heat exchange capacity [1,2]. Together, these mechanisms provide a plausible explanation for the greater thermal asymmetry observed among participants with higher body fat percentages.

Previous thermographic studies have consistently reported an influence of body composition on skin temperature patterns. Salamunes et al. demonstrated that both body fat percentage and fat distribution significantly affected skin surface temperature measured by infrared thermography [7,15]. Similar findings have been reported in studies examining anthropometric characteristics and adiposity-related variables, both at rest and during recovery from physical exercise [16,17]. However, most available evidence has been obtained in athletes, younger adults, or highly selected populations [18,19]. Consequently, information regarding the relationship between body composition and thermal asymmetry in community-dwelling older adults remains limited. The present results extend current knowledge by suggesting that adiposity may be associated not only with skin temperature itself, but also with asymmetry-related thermographic indices in an aging population.

Women presented significantly higher thermal asymmetry values than men. Nevertheless, sex was not independently associated with thermal asymmetry after adjustment for body fat percentage. Interestingly, sex-stratified analyses revealed that the association between body fat percentage and thermal asymmetry was evident in women but not in men [18]. Although the present study was not specifically powered to investigate sex-specific mechanisms, differences in body fat distribution, subcutaneous adipose tissue thickness, and thermoregulatory responses may partially explain these findings. Previous investigations have reported sex-related differences in skin temperature patterns and heat dissipation mechanisms, which are largely attributed to differences in adiposity and regional fat distribution [7,20]. Future studies should further explore potential sex-specific determinants of thermographic asymmetry in older adults.

In contrast to adiposity-related variables, skeletal muscle mass demonstrated a much weaker relationship with thermographic outcomes. Although a modest inverse association was observed between muscle mass and trunk asymmetry, most muscle-related variables were not significantly associated with thermal asymmetry indices. While skeletal muscle plays a fundamental role in metabolic heat production, skin temperature reflects the interaction of multiple physiological processes, including vascular regulation, local perfusion, tissue insulation, and heat transfer dynamics [1,6,18]. Consequently, muscle quantity alone may not adequately explain interindividual differences in resting thermal asymmetry. Moreover, the present study evaluated skeletal muscle quantity using appendicular skeletal muscle mass index (ASMI), which does not capture qualitative characteristics of muscle such as intramuscular fat infiltration, muscle architecture, or contractile function. These qualitative aspects may have a greater influence on heat production and thermoregulatory responses than muscle quantity alone and could partly explain the lack of significant associations observed in the present study. Future studies incorporating direct measures of muscle quality and functional performance are warranted to further clarify the relationship between skeletal muscle and thermographic patterns in older [31] adults. Overall, the study hypothesis was only partially supported. Higher adiposity showed weak but relatively consistent associations with several thermographic asymmetry parameters in the univariate analyses, although these findings should be interpreted cautiously because the adjusted regression model was not statistically significant. In contrast, the hypothesized association between skeletal muscle mass and thermographic asymmetry was not confirmed.

From a practical perspective, these findings highlight the importance of considering body composition when interpreting thermographic assessments in older adults. Infrared thermography is increasingly used as a rapid, non-invasive, and contact-free method for evaluating temperature distribution patterns and physiological responses [8,12,13,14]. However, thermal asymmetry may be influenced not only by clinical or functional conditions but also by adiposity-related characteristics. Failure to account for this source of variability could lead to misinterpretation of thermographic findings when comparing individuals with markedly different body composition profiles. This consideration may be particularly relevant in geriatric populations, where obesity, sarcopenia, and body composition alterations are highly prevalent.

Several limitations should be considered when interpreting these results. First, the cross-sectional design precludes causal inference. Second, participants were recruited from a single geographical area, which may limit external validity. In addition, the study sample included substantially more women than men. This sex imbalance is consistent with the demographic structure of very old populations, in which women substantially outnumber men because of their longer life expectancy [31]. Nevertheless, the smaller number of male participants may have reduced the statistical power and reliability of the sex-stratified analyses. Third, several factors known to influence thermographic outcomes could not be fully controlled. In particular, medication use was not considered in the analyses, despite the high prevalence of vasoactive drugs such as antihypertensive agents and β-blockers in older adults. These medications may influence peripheral blood flow, vasomotor responses, and skin temperature distribution, potentially contributing to variability in the thermographic measurements. Hydration status, physical activity habits, and chronic health conditions may also have influenced the observed thermal patterns. In addition, although wheelchair users were included only if they were able to complete the standardized assessment protocol, the inclusion of participants with different mobility levels may have contributed to additional variability in body composition and thermographic measurements. Finally, although significant associations were identified, the observed correlation coefficients were generally weak, and the regression model explained only a modest proportion of the variability in thermal asymmetry, indicating that additional physiological, clinical, environmental, and behavioral factors—including medication use, hydration status, physical activity, and other potential confounders not included in the model—are likely to contribute substantially to thermographic patterns in older adults. Furthermore, although body fat percentage showed a statistically significant regression coefficient, the overall regression model was not statistically significant, reinforcing the exploratory nature of these findings. In addition, multiple correlation analyses were performed without adjustment for multiple comparisons. Consequently, the possibility of an inflated Type I error rate cannot be excluded, and the findings should therefore be interpreted as exploratory and require confirmation in future studies. Additionally, the TRI and SAP are composite thermographic indices generated automatically by the proprietary ThermoHuman software. Although these indices provide complementary information regarding thermal asymmetry patterns, their underlying computational algorithms are not publicly available and independent external validation remains limited. Therefore, their interpretation should be considered within the context of the ThermoHuman platform used in the present study. Nevertheless, the study was conducted using standardized thermographic procedures and validated body composition assessment methods, providing novel information regarding the relationship between adiposity and thermographic asymmetry in older adults.

Interest in thermography as a tool for evaluating physiological responses in aging populations continues to grow [1,2,8,17]. Understanding the factors that contribute to thermal variability is therefore essential for improving interpretation of thermographic outcomes. The present findings suggest that adiposity should be considered as one of several factors associated with thermographic variability in older adults, rather than as the sole determinant of thermal symmetry patterns. Integrating thermographic assessment with multidimensional evaluations of body composition, functional capacity, and health status may provide a more comprehensive understanding of thermoregulatory function during aging. Future research should investigate whether thermographic variability is associated with clinically relevant outcomes in older adults.

## 5. Conclusions

Body fat percentage showed weak but statistically significant associations with several thermographic asymmetry-related parameters in the univariate analyses, including mean absolute thermal asymmetry, TRI, and SAP. However, these findings should be interpreted cautiously because the adjusted regression model was not statistically significant. In contrast, skeletal muscle mass showed limited associations with thermographic outcomes. Although women exhibited greater thermal asymmetry than men, these differences were attenuated after adjustment for body fat percentage.

Overall, these exploratory findings suggest that adiposity may represent one of several factors associated with variability in thermographic assessments and may therefore be considered when interpreting thermal patterns in older adults. The combined assessment of body composition and thermographic parameters may be useful for future research investigating thermoregulatory function in older adults. Nevertheless, given the weak magnitude of the observed associations and the exploratory nature of the analyses, these findings should be confirmed in larger prospective studies before drawing conclusions regarding their clinical applicability.

## Figures and Tables

**Table 1:** Baseline characteristics of the study participants.

Variable	Total Sample (*n* = 127)
Age, years	81.0 ± 10.7
BMI, kg/m^2^	28.0 ± 5.7
Body fat percentage, %	38.3 ± 11.5
ASMI, kg/m^2^	6.86 ± 1.95
Phase angle, °	3.86 ± 2.19
ECW/TBW ratio	0.403 ± 0.017
Global temperature, °C	33.20 ± 0.97
Trunk thermal asymmetry	0.33 ± 0.16
Upper-limb thermal asymmetry	0.57 ± 0.42
Mean absolute thermal asymmetry	0.46 ± 0.24
TRI	75.3 ± 28.0
SAP	53.3 ± 19.2

Data are presented as mean ± standard deviation (SD). Abbreviations: BMI, body mass index; ASMI, appendicular skeletal muscle mass index; ECW/TBW, extracellular water-to-total body water ratio; TRI, Thermal Recovery Index; SAP, Symmetry Assessment Parameter.

**Table 2:** Sex differences in body composition and thermographic variables.

Variable	Women (*n* = 85)	Men (*n* = 42)	*p*-Value
BMI, kg/m^2^	28.8 ± 6.6	27.1 ± 4.1	0.194
Body fat percentage, %	40.4 ± 11.8	34.3 ± 9.2	<0.001 *
ASMI, kg/m^2^	6.4 ± 1.8	7.7 ± 1.9	<0.001 *
Phase angle, °	3.72 ± 1.17	3.70 ± 1.42	0.929
ECW/TBW ratio	0.402 ± 0.016	0.405 ± 0.015	0.331
Global temperature, °C	33.17 ± 1.05	33.25 ± 0.88	0.660
Trunk thermal asymmetry	0.34 ± 0.14	0.28 ± 0.15	0.006 *
Upper-limb thermal asymmetry	0.55 ± 0.40	0.58 ± 0.47	0.654
Mean absolute thermal asymmetry	0.47 ± 0.22	0.44 ± 0.27	0.045 *
TRI	78.3 ± 25.5	67.4 ± 30.9	0.044 *
SAP	56.1 ± 17.7	47.4 ± 20.2	0.008 *

Values are presented as mean ± standard deviation for descriptive purposes. Group comparisons were performed using the Mann–Whitney U test due to non-normal distribution of the variables. * *p* < 0.05.

**Table 3:** Spearman correlations between thermographic parameters and body composition variables.

Thermographic Variable	BMI	PBF	SMM	ASMI	PhA	ECW/TBW
Global temperature	−0.137 (0.144)	−0.131 (0.165)	−0.095 (0.317)	0.011 (0.906)	−0.070 (0.464)	**0.260 (0.005)**
Trunk thermal asymmetry	0.052 (0.580)	0.171 (0.071)	**−0.199 (0.035)**	−0.138 (0.140)	−0.013 (0.892)	−0.080 (0.400)
Upper-limb thermal asymmetry	0.161 (0.085)	**0.229 (0.015)**	−0.055 (0.561)	−0.074 (0.431)	−0.001 (0.995)	0.106 (0.264)
Mean absolute thermal asymmetry	0.162 (0.083)	**0.280 (0.003)**	−0.164 (0.082)	−0.129 (0.169)	−0.015 (0.876)	0.072 (0.451)
TRI	0.167 (0.074)	**0.250 (0.008)**	−0.140 (0.138)	−0.093 (0.320)	−0.039 (0.680)	0.054 (0.567)
SAP	**0.198 (0.033)**	**0.276 (0.003)**	−0.141 (0.135)	−0.109 (0.243)	−0.013 (0.893)	−0.001 (0.988)

Values are Spearman’s rank correlation coefficients (ρ) with *p*-values in parentheses. Statistically significant associations (*p* < 0.05) are shown in bold. Pairwise deletion was used; sample size varied according to variable availability. Abbreviations: BMI, body mass index; PBF, percent body fat; SMM, skeletal muscle mass; ASMI, appendicular skeletal muscle mass index; PhA, phase angle; ECW/TBW, extracellular water-to-total body water ratio; TRI, Thermal Recovery Index; SAP, Symmetry Assessment Parameter.

**Table 4:** Multiple linear regression analysis examining the association between body fat percentage and mean absolute thermal asymmetry.

Independent Variable	B	SE	β	t	*p*	95% CI for B
Constant	0.387	0.219	—	1.768	0.080	−0.047 to 0.820
PBF, %	0.005	0.002	0.224	2.252	0.026 *	0.001 to 0.009
Age, years	−0.001	0.002	−0.056	−0.563	0.574	−0.006 to 0.003
Sex	−0.008	0.050	−0.016	−0.159	0.874	−0.107 to 0.091

Model statistics: R = 0.223; R^2^ = 0.050; adjusted R^2^ = 0.050; F (3106) = 1.840; *p* = 0.144. Dependent variable: mean absolute thermal asymmetry (ASYMMETRY_ABS_AVG). Sex was coded as 1 = women and 2 = men. Abbreviations: PBF, percent body fat; SE, standard error; β, standardized regression coefficient; CI, confidence interval. Statistically significant associations (*p* < 0.05) are indicated by an asterisk.

## Data Availability

The datasets generated and analyzed during the current study are not publicly available due to privacy and ethical restrictions related to the protection of participant data. De-identified data may be made available by the corresponding author upon reasonable request and subject to approval by the relevant ethics committee, in accordance with institutional policies and applicable data protection regulations.
